# Scarlet Fever

**Published:** 1900-12-22

**Authors:** Percival Mackie

**Affiliations:** Resident Medical Officer, Bristol City Isolation Hospitals, Ham Green


### Hospital Clinics and Medical Progress.

### SCARLET FEVER.

By Percival Mackie, M.R.C.S.Eng., L.R.C.P.Lond.; Resident Medical Officer, Bristol City Isolation Hospitals, Ham Green.

The following remarks are based on the experience gained from the observation of the first 700 cases admitted to this hospital.

Scarlet fever is a disease generally diagnosed with ease, often quite unmistakable and usually mild; few diseases, however, vary so much in intensity, and few are more difficult to distinguish when anomalous.

Generally a disease marked by mild fever, sore throat, the presence of a scarlet rash followed by peeling, it may, on the one hand, be so mild that only one symptom exists, and that only for a few hours, whilst, on the other, it can be so severe that death is inevitable from the first. The average case requires no medicinal treatment throughout, and often not even local application to the throat. The severest cases are not affected by medicine, no drug having any effect in modifying the course of the disease. The seasonal variation in its prevalence is marked, the minimum being in April and the maximum in October.

It is essentially a disease of childhood, the large majority of cases being under 15 years of age. In the present series of cases 35 per cent. were under 5 years, 77 per cent. were under 10 years, and 90 per cent. under 15 years. The fatality, again, is higher in children, especially under 5 years; broadly the younger the child the more serious the disease. Thus out of 17 deaths 14 were under 5 years, although the percentage of admissions under 5 was only 35.

*Pathology and Morbid Anatomy.*—Everything points to the probability of scarlet fever being due to a micro-organism. So far, however, its existence has not been proved. The constancy of the throat symptoms suggests strongly that the throat is the place of entrance of the contagion. Dr. W. Dowson has suggested the very ingenious and plausible theory that the faucial condition in this disease bears the same relation to the rash and secondary symptoms as does the chancre of syphilis to the rash, glandular enlargements, and other manifestations of that disease.

Cultures from the throat always show plenty of pyogenic organisms, but none with greater constancy than a short streptococcus. This has been looked upon as the cause of scarlet fever; whether rightly or wrongly it is impossible to say; but we must remember that neither from a cultural nor from a morphological nor yet from an experimental point of view can it be distinguished from the streptococcus found in such divergent conditions as erysipelas and endocarditis, puerperal peritonitis, and boils. In a recent paper Dr. Class[1] has described a large diplococcus isolated from cases of the disease with great constancy and believed by him to be the long-sought-for scarlet fever organism.

The most frequent cause of death, directly or indirectly, is a severe throat condition—the type of the disease known as scarlatina anginosa. In 17 deaths it has been the chief cause in 10. In these 10 cases this anginose condition was associated with deep-seated suppuration in the neck in two cases, with œdema of the larynx, requiring tracheotomy, followed by septic pneumonia in three cases, with septicæmia or pyæmia in three cases, with suppurative meningitis in one case and in the tenth case beyond the ulcerated fauces no other complication existed. The remaining seven deaths were due to the following conditions:—

Acute nephritis, two cases.
Septicæmia, two cases.
Gastro-enteritis c. ulcerative colitis, one case.
Persistent vomiting, one case.
Diphtheria, one case.

There are no marked post-mortem signs in those cases which die from anginose scarlet fever *per se*; death seems to be due to an overwhelming dose of toxin absorbed from the ulcerated throat. Streptococci are often found pervading the tissues in small numbers, and may generally be recovered from the blood after death. The organs are dark, soft, and stained with blood; the spleen is slightly enlarged; and cloudy swelling or early fatty degeneration in various organs are almost constant signs. Very extensive ulceration is often found in the fauces, sometimes involving the whole naso- and oro-pharynx, together with widespread œdema and inflammation of all surrounding structures. Sometimes extensive necrosis of the laryngeal cartilages is met with. Septic pneumonia arises from inspiration of discharges from the throat, and this often happens when the child becomes partly unconscious. Most of these serious complications result from the direct extension of suppuration from the throat.

Nephritis is probably due to the excretion of poisons by the kidneys, for although organisms are found in the inflamed kidneys they do not occur with sufficient constancy to warrant their being looked upon as the direct cause of the inflammation. In all suppurations arising during the course of the disease streptococci generally associated with staphylococci are found; but there is no evidence to show that they are different from those found in other diseases.

*Incubation.*—The incubation stage is said to be invariably under a week, and more often to be 24 or 36 hours. We have seen one or two cases where the symptoms did not develop until between two and three weeks after admission into the general wards. It is probable, however, that these children may have escaped actual infection until within a few days of the eruption of the rash. In some epidemics and in the fulminant cases the incubation stage may be reduced to a few hours.

*Invasion.*—General malaise is the first symptom, often accompanied by vague pains in the limbs, back, and head. Some time during the same day the throat feels stiff and sore, and a few hours after that the rash appears. Vomiting and sleeplessness are often troublesome symptoms, particularly in adults. When delirium is met with in young children it is a bad sign.

*The Rash.*—The rash appears about 12 to 24 hours after the onset of symptoms, and generally begins on the upper part of the chest and neck and then spreads to the abdomen, back, arms, and legs. We have never seen the face or scalp affected, but on several occasions have noticed a well-marked punctate rash on the palms and soles, although by some it is stated never to occur in any of these situations. It is usually most vivid on the neck and in the axillæ and groins.

When typical it shows two well-marked elements: one a simple, general erythema, which seems to lie deeply; the other element is apparently superimposed on the first-named, and appears as minute red points of a darker shade, and apparently more superficial, scattered all over the skin. The general blush is due to injection of subcuticular vessels and the punctate element to injection of the small vessels in the papillæ of the skin.

[1] Lancet, July 14th, 1900.

*(To be continued.)*

